# The Effect of the NF*κ*B-USP9X-Cx43 Axis on the Dynamic Balance of Bone Formation/Degradation during Ossification of the Posterior Longitudinal Ligament of the Cervical Spine

**DOI:** 10.1155/2022/1604932

**Published:** 2022-03-29

**Authors:** Xiaoqiu Yuan, Yongfei Guo, Jilu Liu, Jingchuan Sun, Lei Shi, Jinhao Miao, Jiangang Shi, Yu Chen

**Affiliations:** ^1^Spine Center, Department of Orthopaedics, Changzheng Hospital, Second Military Medical University, China; ^2^Naval Hospital of Eastern Theater, PLA, China

## Abstract

Connexin 43- (Cx43-) mediated nuclear factor kappa-light-chain-enhancer of activated B cell (NF-*κ*B) signaling has been found involved in the ossification of the posterior longitudinal ligament (OPLL). However, the underlying mechanism how OPLL is regulated has not been elucidated. In the present study, primary ligament fibroblast were isolated; immunoprecipitation (IP) and liquid chromatography-mass spectrometry (LC-MS) assays were applied to identify potential binding proteins of Cx43. Protein interaction was then confirmed by co-IP assay. Alkaline phosphatase (ALP) activity and alizarin red staining were used to evaluate ossification. Luciferase reporter assay and chromatin immunoprecipitation (ChIP) assay were employed to assess the binding between NF-*κ*B p65 and target gene. Lipoxygenase inhibitor (5,8,11-eicosatriynoic acid, EPA) was applied to induce endoplasmic reticulum (ER) stress, and 4-phenylbutyrate (4-PBA) was used as an ER-stress inhibitor. Expression of USP9X, Cx43, and nuclei p65 in ligaments from patients and controls was detected by Western blotting. The results showed that ubiquitin-specific protease 9 X-chromosome (USP9X), a deubiquitylating enzyme, was a candidate of Cx43 binding proteins, and USP9X inhibited Cx43 ubiquitination. In vitro experiments showed that USP9X promoted ossification of primary ligament fibroblasts and nuclear translocation of NF-*κ*B p65 by regulating Cx43 expression. Moreover, NF-*κ*B can bind to the USP9X promoter to promote its transcription. When ER stress was inhibited by 4-PBA, USP9X levels, NF-*κ*B nuclei translocation, and ALP activity were decreased. Reverse results were obtained when ER stress was induced by EPA. PDTC, an NF-*κ*B inhibitor, could abolish the effects of EPA. Furthermore, USP9X, Cx43, and nuclei p65 were significantly upregulated in ligaments from OPLL patients than non-OPLL controls. USP9X was positively correlated with CX43 and nuclei p65 in OPLL samples. Overall, the findings suggest that the ER stress–NF*κ*B-USP9X-Cx43 signaling pathway plays an important role in OPLL progression.

## 1. Introduction

Ossification of the posterior longitudinal ligament (OPLL), first reported in 1838, is a disease characterized by progressive ectopic ossification of the spine's posterior longitudinal ligament [1, 2]. OPLL is influenced by geographic location and ethnicity, with a higher incidence observed in the East Asian population [3]. Its prevalence in Japan ranges from 1.9% to 4.3%, and 12-year prevalence in Korea was 2.04% [1, 4]. Studies have also shown a prevalence of OPLL of 1.3% in Caucasian Americans and 4.8% in Asian Americans [5]. Surgery is currently the only effective treatment option [6]. Nevertheless, it is a relatively difficult and high-risk operation under certain situations and might be associated with serious complications such as spinal cord injury. Therefore, its pathogenesis and surgical treatment are important concerns.

OPLL typically begins with mild or no symptoms at all. OPLL slowly progresses and may eventually cause spinal stenosis [7, 8]. The gradual replacement of ligament tissue with bony components may also cause spinal cord or spinal cord blood vessel compression, leading to radiculomyelopathy [1, 9]. Many factors, including hormones, cytokines, and growth factors, affect the ossification process [10, 11]. For example, bone morphogenetic protein (BMP)-2 [12] and transforming growth factor (TGF)-*β* [13] are important initiators of the ossification process in OPLL. Studies of its pathogenesis suggest that OPLL is a multifactorial disease. Nongenetic and genetic factors contribute to its development [2]. For example, age, a high body mass index, and exercise are independent OPLL risk factors [14–16]. COL11A2, the TGF-*β*3 polymorphisms of rs226862 and rs22847, and BMP4 SNPs rs17563(C/T) and rs76335800 (A/T) are associated with OPLL [17–20]. However, previous studies demonstrate continued OPLL ossification in nonsurgical and surgically treated patients. Hence, understanding the regulatory mechanism of the osteogenesis process might be the key to slow OPLL progression.

Connexin 43 (Cx43), a gap-junction protein, is upregulated in OPLL tissue or OPLL fibroblasts. It plays a key role in ligament fibroblasts osteogenesis [21–24]. Our group proved that Cx43-mediated nuclear factor kappa-light-chain-enhancer of activated B cell (NF-*κ*B) (p65) signaling plays an important role in mechanical stress-induced OPLL and ligament fibroblast inflammation [21, 24, 25]. The excessive accumulation of unfolded proteins, known as the endoplasmic reticulum (ER) stress response, is important in osteogenesis [26, 27]. Our previous study showed that mechanical stress could activate ER stress [22]. ER stress is a vital player in the incidence of OPLL, and it participates in Cx43-related OPLL [21]. With these previous findings, we wanted to investigate the role of Cx43 in OPLL further. Using mass spectrometry, we first identified the binding proteins of Cx43. Ubiquitin-specific proteases 9 X-chromosome (USP9X), also known as fat facets in mouse (FAM), was identified as a Cx43-binding protein. USP9X, a deubiquitylating enzyme, removes monoubiquitin and polyubiquitin chains from a substrate [25]. USP9X is closely linked to the TGF signaling pathway, which is related to bone formation [26]. Laing et al. reported that Cx43 is degraded via the ubiquitin-proteasome pathway [27]. Thus, we studied the regulatory effect of USP9X on Cx43 expression. We also investigated the underlying mechanism by which USP9X-Cx43 regulates OPLL development.

## 2. Materials and Methods

### 2.1. Patient Information

The Research Ethics Committees of Changzheng Hospital (Shanghai, China) approved the protocol (Approval number: 2019SLYS2; Date:Aug-31-2019). Ten non-OPLL patients with cervical spine trauma and 25 OPLL patients with anterior cervical decompression surgery were included. X-rays, computed tomography scans, and magnetic resonance imaging were used for OPLL diagnosis. Informed consent was obtained from each patient.

### 2.2. Primary Ligament Fibroblast Culture

Primary ligament fibroblast culture was performed with a previously reported method [21]. Briefly, nonossified ligament tissues collected during surgery were carefully dissected to avoid contamination of osteogenic cells. The tissue was cut into 0.5 mm^3^ pieces, washed five times with phosphate-buffered saline (PBS), and cultured in Dulbecco's modified Eagle's medium (DMEM, Gibco, Waltham, MA, USA) with 10% fetal bovine serum (Gibco). Derived cells were digested with trypsin and further cultured in a humidified CO_2_ incubator (5%); 12/15-lipoxygenase inhibitor (5,8,11-eicosatriynoic acid, EPA, Sigma-Aldrich, Shanghai, China) was used to induce ER stress at a concentration of 20 nM [21]. 4-Phenylbutyrate (4-PBA, Cayman, Ann Arbor, MI) was used as an ER stress inhibitor at a concentration of 10 nM [21].

### 2.3. Establishment of Stable Cell Lines

The Cx43 gene with a 3× Flag tag at its 5′-end was amplified by PCR and inserted into a GV348 lentiviral vector (Genechem, Shanghai, China). To prepare the recombinant lentivirus, we used the pHelper 1.0 plasmid (15 *μ*g, GeneChem), pHelper 2.0 plasmid (10 *μ*g, GeneChem), and the Cx43-overexpression plasmid or vector (20 *μ*g) to transfect HEK293T using Lipofectamine-2000 (Invitrogen, Shanghai, China). The medium was replaced with a complete medium 6 hours later. Forty-eight hours after transfection, the recombinant lentivirus was collected, concentrated, quantified (5 × 10^8^ TU/ml), and used to transduce primary ligament fibroblasts. Twenty-four hours after transduction, puromycin (2 *μ*g/ml, Sigma-Aldrich) was used to select cells that were stably transfected. Overexpression of Cx43 was confirmed by Western blotting.

### 2.4. Lysate Preparation and Western Blotting

Lysates were made using a radio-immunoprecipitation assay buffer with proteinase inhibitors (Sigma, Shanghai, China). According to the manufacturer's protocol, the cytosolic or nuclear parts were made using a nuclear/cytosol fractionation kit (Biovison, Milpitas, CA). Proteins were separated by sodium dodecyl sulfate-polyacrylamide gel electrophoresis (SDS-PAGE) and transferred to PVDF membranes (Bio-Rad, Philadelphia, PA). Membranes were blocked with 3% BSA, incubated overnight at 4°C with primary antibodies (Table S1), washed, and incubated with HRP-conjugated rabbit secondary antibody (Beyotime, Shanghai, China) at room temperature for 1 hour. Protein bands were detected using enhanced chemiluminescence (Amersham Biosciences, Marlborough, MA) with a LAS400 image analyzer (FujiFilm, Stamford, CT).

### 2.5. Immunoprecipitation (IP) and Liquid Chromatography-Mass Spectrometry (LC-MS) Assay

Proteins extracted from stable cells were first incubated with IgG and protein A-G beads (Santa Cruz Bio., Dallas, TX) at 4°C for two hours, then incubated with anti-Flag antibody (Sigma, Shanghai, China) overnight at 4°C. Immunoprecipitated-complexes were eluted with Flag peptides (Sigma-Aldrich), separated by SDS-PAGE, and stained by Coomassie Brilliant Blue. Various bands were excised, digested by trypsin, and analyzed using LC-MS [28].

### 2.6. Coimmunoprecipitation (Co-IP) Assays

Co-IP assays were performed as previously described [29]. Cell extraction was incubated with anti-USP9X antibody, anti-Cx43 antibody (Abcam, Shanghai, China), or control IgG (Santa Cruz Bio, Dallas, TX) for one hour at 4°C, followed by incubation with A/G-agarose for three hours at 4°C. Precipitates were washed three times with lysis buffer, and proteins were detected by Western blotting.

### 2.7. RNA Isolation and Quantitative RT-PCR

RNA was extracted with Trizol (Invitrogen) following the manufacturer's protocol. mRNA levels of the indicated genes were measured by RT-PCR [29] using PCR master mix (Thermo, Shanghai, China) on a CFX96 qPCR instrument (Bio-Rad, Philadelphia, PA) with the following conditions: 96°C 10 min, 42 cycles of 94°C 15 s, 58°C 40 s. The relative fold-change was determined by 2^−△△CT^. All experiments were repeated three times. Table S2 lists the primers.

### 2.8. Lentivirus Preparation

Short hairpin RNA (shRNA) oligos targeting USP9X (Table S3) were ligated into the pLKO.1 vector digested by AgeI and EcoRI (Addgene, Beijing, China). USP9X was ligated into pLVX-puro (Clontech, Mountain View, CA). The plasmids mentioned above, with psPAX2 and pMD2.G plasmids, were then used to transfect 293T cells to produce lentivirus [30].

### 2.9. ALP Activity Assay

ALP activity was detected with a commercial kit (Jiancheng Bio, Nanjing, China) according to the manufacturer's protocol. The cell lysate was incubated with 1 mg/ml 4-nitrophenyl phosphate for 30 min; absorbance was determined at OD 405 using a microplate reader (Bio-Tek, Winooski, VT).

### 2.10. Alizarin Red Staining

Osteogenic differentiation medium was added to 80% confluent ligament fibroblasts in the logarithmic growth phase. The medium was changed every two days. After 21 days, cells from different treatment groups were washed with PBS 2 to 3 times, fixed with 4% formaldehyde for 30 min, washed again with PBS 2 to 3 times, and stained with Alizarin Red S solution for 3–5 minutes (Sigma-Aldrich) as previously described [31]. The Alizarin red-stained area was quantified with ImageJ software (NIH, USA).

### 2.11. Luciferase Reporter Assay

The full-length promoter region of USP9X was introduced into pGL3 plasmids (Promega, San Luis Obispo, CA), transfected into primary ligament fibroblast cells using lipofectamine 2000 (Invitrogen, Carlsbad, CA), and treated as indicated. Luciferase activity was measured with a Luciferase assay kit (Promega) following the manufacturer's protocol [32].

### 2.12. Chromatin Immunoprecipitation (ChIP) Assay

A ChIP assay [33] was carried out using cells treated by either Tumor necrosis factor-alpha (TNF-*α*, R&D) (10 ng/ml) or pyrrolidine dithiocarbamate (PDTC, Selleck) (10 *μ*M) for 24 h; 1% formaldehyde was used for chromatin crosslinking. Subsequent chromatin solutions were incubated with either anti-p65 antibody (Cell Signaling Technology) or IgG overnight at 4°C with rotation. The binding of the USP9X promoter was measured using PCR with the primers: 5′TGTTTGAAGGGCTCCTATGG3′ and 5′GGGAAGACAATGCGGTAAAG3′.

### 2.13. Statistical Analysis

Graphpad Prism 6.0 (La Jolla, CA) was used for the statistical analysis. Comparisons were made by Student's *t*-test and analysis of variance (ANOVA). A *P* value < 0.05 was significant.

## 3. Results

### 3.1. USP9X Interacted with and Inhibited Cx43 Ubiquitination in Primary Ligament Fibroblast Cells

**Figure 1:**
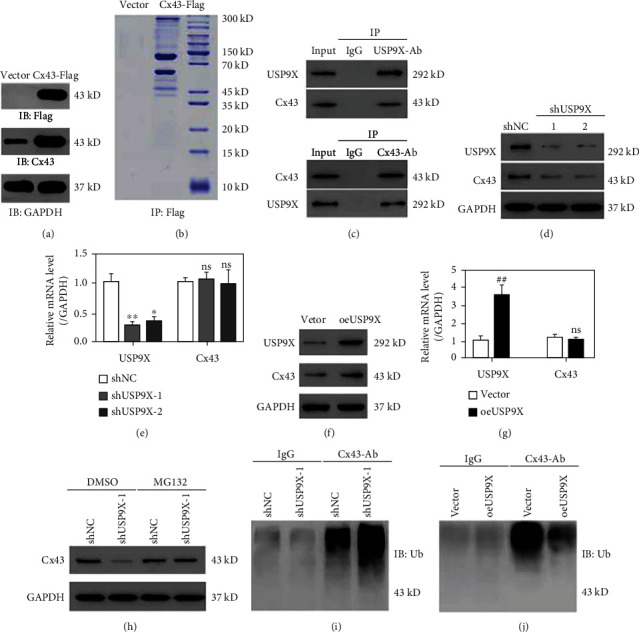
Cx43 interacted with USP9X and USP9X inhibited Cx43 ubiquitination. (a) Cx43 expression level from ligament fibroblast cells stably transfected with Cx43-Flag or vector alone was detected by Western blot using anti-Flag or anti-Cx43 antibodies. (b) Proteins precipitated using anti-Flag antibodies were resolved by SDS-PAGE and stained using Coomassie Brilliant Blue. (c) Western blots of proteins precipitated using anti-USP9X antibodies and anti-Cx43 antibodies. (d) Western blots showing that USP9X was successfully knocked down at the protein level. USP9X KD caused Cx43 downregulation. (e) qPCR results showing that USP9X was successfully knocked down at the mRNA level, and no effect was observed on Cx43 expression. (f) Western blots showing that USP9X was successfully overexpressed at the protein level, and USP9X overexpression caused Cx43 upregulation. (g) qPCR results showing that USP9X was successfully overexpressed at the mRNA level, and no effect was observed on Cx43 expression. (h) Western blots showing that primary ligament fibroblast treatment silenced USP9X with MG132 (10 *μ*M) for 4 h significantly upregulating Cx43 expression. (i) Effects of USP9X silencing on Cx43 ubiquitination. (j) Effects of USP9X overexpression on Cx43 ubiquitination. ^∗^*P* < 0.05, ^∗∗^*P* < 0.01 vs. shNC. ^##^*P* < 0.01 vs vector. Ns: not significant.

To study the binding protein of Cx43, ligament fibroblast cells stably expressed Cx43-Flag were established. Western blot results confirmed the overexpression of Cx43-Flag (Figure 1(a)). Then, protein extracts from these cells were incubated with anti-Flag beads, eluted, and resolved on SDS-PAGE. Various bands were excised, trypsinized, and analyzed using LC-MS (Figure 1(b)). Proteomic results revealed a variety of Cx43 binding proteins, including USP9X (Table S4). Next, a Co-IP assay was performed with antibody against Cx43 or USP9X. The results confirmed that endogenous Cx43 interacted with endogenous USP9X (Figure 1(c)).

Considering that USP9X is a well-known deubiquitylating enzyme [25], we then explored whether USP9X affected Cx43 expression. USP9X was knocked down (KD) or overexpressed in primary ligament fibroblasts (Figure S1). The results showed that USP9X knockdown (KD) decreased the Cx43 protein level, while USP9X overexpression enhanced the CX43 protein level. However, USP9X overexpression or KD did not affect Cx43 expression at the mRNA level (Figures 1(d)–1(g)). USP9X KD caused a Cx43 protein decrease that was abolished by the MG132 supplement (Figure 1(h)), suggesting proteasome involvement in Cx43 downregulation. Moreover, Cx43 ubiquitination was increased by USP9X KD, and decreased by USP9X overexpression (Figures 1(i) and 1(j)). Together, these findings indicate that USP9X interacts with Cx43 and may serve as a deubiquitinase of Cx43.

### 3.2. USP9X Expression Promoted the Ossification of Primary Ligament Fibroblasts

**Figure 2:**
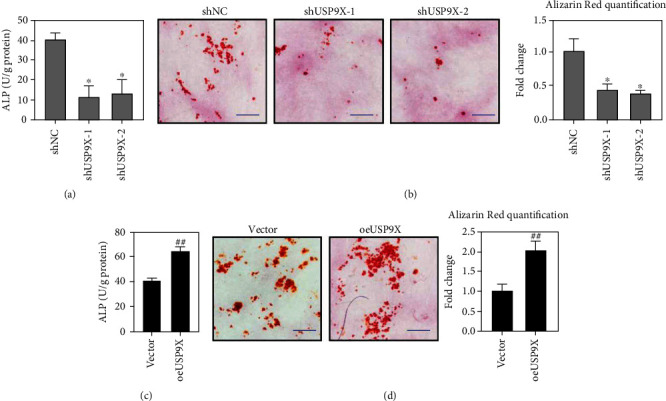
USP9X expression affected the ossification of primary ligament fibroblasts. USP9X KD inhibited the ossification of primary ligament fibroblasts indicated by ALP assay (a) and Alizarin Red staining (b). USP9X overexpression promoted the ossification of primary ligament fibroblasts indicated by ALP assay (c) and Alizarin Red staining (d). Scale bar: 50 *μ*m. ^∗^*P* < 0.05 vs. shNC. ^##^*P* < 0.01 vs. vector.

To investigate the effect of USP9X expression on the ossification process, ALP activity and alizarin red staining were applied in primary ligament fibroblasts with USP9X knockdown or overexpression. The results suggested that USP9X KD significantly decreased ALP activity and the intensity of alizarin red staining (Figures 2(a) and 2(b)). USP9X overexpression enhanced ALP activity and the intensity of alizarin red staining (Figures 2(c) and 2(d)), suggesting that USP9X promoted the ossification process of primary ligament fibroblasts.

### 3.3. USP9X Regulated Ossification via Cx43

**Figure 3:**
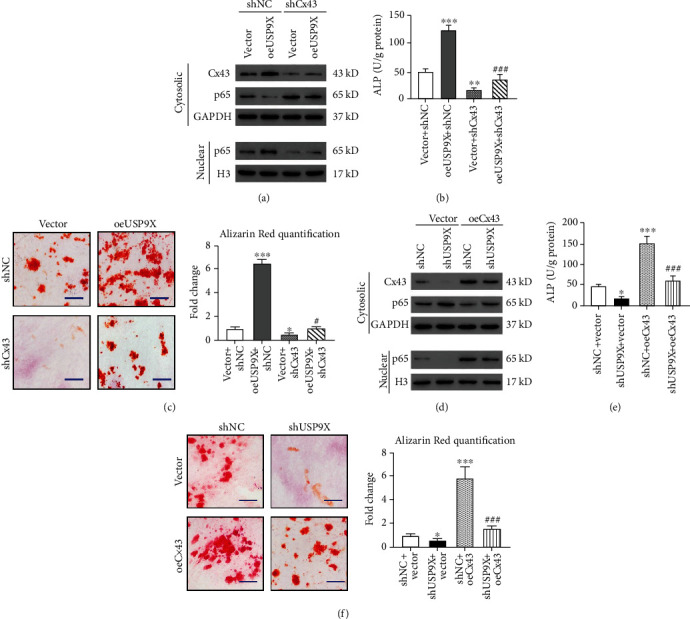
USP9X regulated Cx43-associated ossification of primary ligament fibroblasts. (a) Overexpression of USP9X in ligament fibroblasts resulted in Cx43 upregulation, p65 reduction in the cytosol, and p65 accumulation in nuclei, which were reversed by Cx43 KD in ligament fibroblasts overexpressing USP9X. (b) Overexpression of USP9X increased ALP activity which was significantly reduced by Cx43 KD. (c) USP9X KD in ligament fibroblasts resulted in a Cx43 decrease, p65 accumulation in the cytosol, and p65 reduction in nuclei, which were reversed by Cx43 overexpression. (d) USP9X KD decreased ALP activity which was significantly enhanced by Cx43 overexpression. Scale bar: 50 *μ*m. ^∗^*P* < 0.05, ^∗∗^*P* < 0.01, ^∗∗∗^*P* < 0.001 vs. shNC + Vector; ^###^*P* < 0.001 vs. shUSP9X + Vector.

To know how USP9X regulates the ossification process, we checked Cx43 and NF-*κ*B signaling. Western blot showed that USP9X overexpression upregulated CX43 expression, p65 accumulation in nuclei, ALP activity, and alizarin red staining intensity, while Cx43 KD could reverse the effects of USP9X overexpression (Figures 3(a)–3(c)). Cx43 overexpression also reversed USP9X KD, which caused a reduction in Cx43 expression, nuclear accumulation of p65, ALP activity, and alizarin red staining intensity (Figures 3(d)–3(f)). These data suggest that Cx43 plays an important role in ossification and USP9X-regulated ossification via Cx43.

### 3.4. USP9X Transcription Was Regulated by NF-*κ*B p65

**Figure 4:**
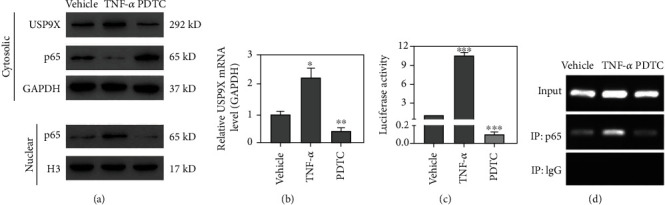
NF-*κ*B p65 mediated the transcription of USP9X in primary ligament fibroblasts. (a) Ligament fibroblasts were treated with either TNF-*α* (10 ng/ml) or PDTC (10 *μ*M) for 24 h. Western blots showed that TNF-*α* treatment caused a significant USP9X increase, p65 reduction in the cytosol, and p65 accumulation in nuclei. PDTC treatment caused a significant USP9X decrease, p65 accumulation in the cytosol, and p65 reduction in nuclei. (b) qPCR results showed that TNF-*α* treatment caused a significant USP9X increase at the mRNA level, while PDTC treatment resulted in significant downregulation of USP9X. (c) USP9X promoter transcription activity was enhanced by TNF-*α* treatment but decreased by PDTC treatment. (d) ChIP results showed that NF-*κ*B p65 binds to the USP9X promotor. ^∗^*P* < 0.05, ^∗∗^*P* < 0.01, ^∗∗∗^*P* < 0.001 vs. vehicle.

To study the potential association between NF-*κ*B and USP9X, TNF-*α* (10 ng/ml) or PDTC (10 *μ*M) was used as an NF-*κ*B activator or inhibitor to treat ligament fibroblasts. Results showed that TNF-*α* increased nuclear accumulation of NF-*κ*B p65, USP9X mRA and protein expression, and transcription activity of USP9X promoter. Administration of PDTC resulted in opposite phenomena (Figures 4(a)–4(c)). The ChIP assay further confirmed the binding of NF-*κ*B p65 to the promoter region of USP9X (Figure 4(d)). These findings indicate that NF-*κ*B can bind to the USP9X promoter to promote its transcription.

### 3.5. ER Stress Involved in the NF-*κ*B/USP9X-Mediated Ossification

**Figure 5:**
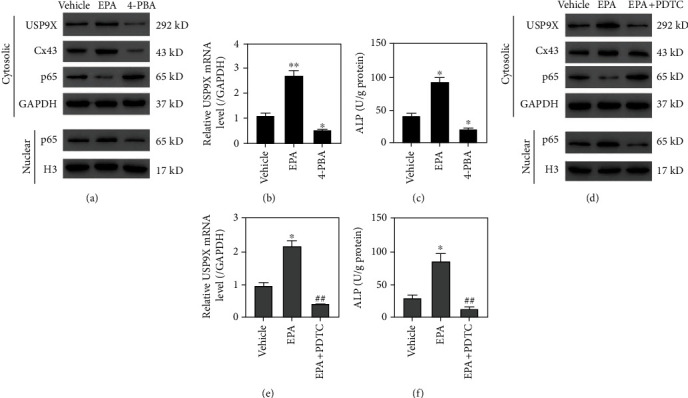
ER stress involved in NF-*κ*B/USP9X-mediated ossification of primary ligament fibroblasts. EPA (10 *μ*M), 4-PBA (2 mM), or EPA plus PDTC (10 *μ*M) was used to treat primary ligament fibroblasts, and Western blots showed that EPA treatment caused a significant USP9X increase, a Cx43 increase, a p65 decrease in the cytosol, and a p65 increase in nuclei. In contrast, 4-PBA treatment led to a significant USP9X decrease, a Cx43 decrease, a p65 increase in the cytosol, and a p65 decrease in nuclei (a). qPCR results showed that EPA treatment caused a significant USP9X increase while 4-PBA treatment caused a significant decrease of USP9X at the mRNA level. (c) EPA treatment caused a significant increase in ALP activity, while 4-PBA treatment caused a significant decrease in ALP activity. (d) Western blots showed that PDTC treatment reversed the EPA-induced USP9X increase, Cx43 increase, p65 decrease in the cytosol, and p65 increase in nuclei. (e) PDTC treatment reversed the EPA-induced USP9X increase at the mRNA level. (f) PDTC treatment reversed the EPA-induced increase of ALP activity. ^∗^*P* < 0.05, ^∗∗^*P* < 0.01 vs. vehicle; ^##^*P* < 0.01 vs. EPA.

To check whether ER stress is involved, primary ligament fibroblasts were treated by EPA (10 *μ*M) or 4-PBA (2 mM). Results showed that induction of ER stress by EPA increased USP9X at the protein and mRNA levels, promoted NF-*κ*B nuclei translocation, and enhanced ALP activity. In contrast, inhibition of ER stress by 4-PBA decreased USP9X, inhibited NF-*κ*B nuclei translocation, and reduced ALP activity (Figures 5(a)–5(c)). Furthermore, EPA-induced USP9X expression, NF-*κ*B nuclei translocation, and ALP activity were abolished by NF-*κ*B signaling inhibition with the administration of PDTC (Figures 5(d)–5(f)). Together, these data indicate that ER stress is involved in the NF-*κ*B/USP9X-mediated ossification process.

### 3.6. USP9X Was Significantly Upregulated in Ligament Tissues from OPLL Patients

**Figure 6:**
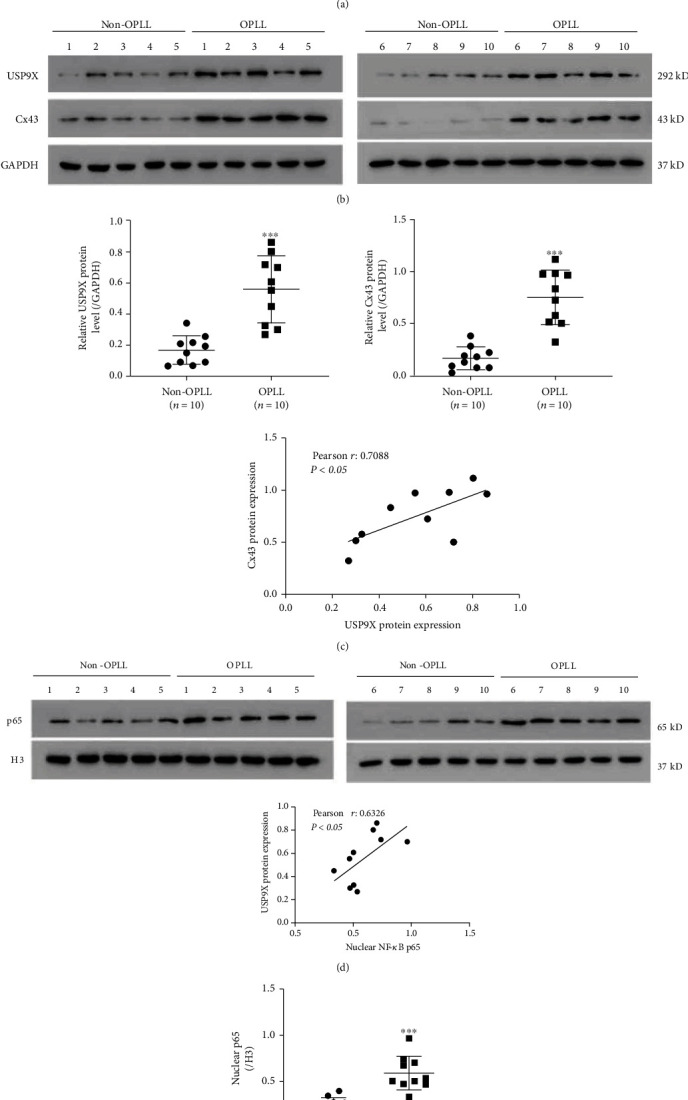
USP9X was significantly upregulated in ligaments of OPLL patients. (a) qPCR results indicated that USP9X mRNA was sharply increased in the OPLL group compared to the non-OPLL group. (b, c) Western blots showed that USP9X and Cx43 were significantly elevated in the OPLL group (*n* = 10) compared to the non-OPLL group (*n* = 10). A Pearson correlation analysis showed that USP9X was positively correlated with CX43. (d, e) Western blots showed that nuclei p65 was significantly elevated in the OPLL group (*n* = 10) compared to the non-OPLL group (*n* = 10). A Pearson correlation analysis showed that USP9X was positively correlated with nuclei p65.

Next, clinical ligament tissues from OPLL patients were collected to investigate the role of USP9X in OPLL. Results (Figure 6(a)) showed that USP9X mRNA was significantly upregulated in ligaments from the OPLL group (*n* = 25) compared to the non-OPLL group (*n* = 10). Western blot analysis was then performed in 10 available OPLL samples and 10 non-OPLL samples. This analysis showed that USP9X protein, Cx43 protein, and nuclei p65 were significantly upregulated in ligaments of the OPLL group compared to the non-OPLL group (Figures 6(b)–6(e)). A Pearson correlation analysis showed that USP9X was positively correlated with CX43 (Figure 6(c)) and nuclei p65 (Figure 6(d)) in OPLL samples.

## 4. Discussion

Cx43-mediated NF-*κ*B (p65) signaling plays an important role in mechanical stress-induced OPLL. However, its regulatory mechanism has not been elucidated. In this study, we further dissected Cx43-mediated NF-*κ*B (p65) signaling and revealed that ER stress and USP9X also play an important role in the development of OPLL.

Evidence has suggested that the deubiquitylating enzyme USP9X is able to regulate osteoblast function and bone formation through regulating TGF*β*/BMP signaling. In the current study, we found that USP9X overexpression promoted the ossification of primary ligament fibroblasts, which further confirmed the functions of USP9X in bone cells. It is reported that USP9X hydrolyzes the monoubiquitination of Smad4, which serves as the central mediator of TGF*β* signaling [26, 34]. USP9X interacts with Smurf1 and stabilizes it [35], which is a negative regulator in TGF*β*/BMP signaling pathway and plays a key role in osteogenic differentiation and bone formation [36, 37]. Data also showed that Cx43 is degraded via the ubiquitin-proteasome pathway [27]. In this study, using gene manipulation technology, Western blot, and qPCR analysis, we showed that USP9X expression regulated the protein level of Cx43 but had no effect on its mRNA level. Further, IP assay showed that USP9X interacted with Cx43 and suppressed Cx43 ubiquitination. These data suggest that USP9X may deubiquitylate Cx43. Moreover, Cx43 promotes OPLL [21], and we found that USP9X affects the ossification process of primary ligament fibroblasts through regulating Cx43. NF-*κ*B signaling controls DNA transcription, cytokine secretion, and cell survival [38]. Our previous study has shown that NFкB (p65) was activated in OPLL patients, and activation of NF*κ*B (p65) signal was dependent on Cx43 [39]. In the current study, we found that USP9X overexpression promoted nuclear translocation of NF-*κ*B p65 by regulating Cx43 expression. Moreover, NF-*κ*B p65 can bind to the USP9X promoter to promote its transcription. Thus, USP9X, Cx43, and NF-*κ*B p65 formed a feedback loop to modulate the ossification process of primary ligament fibroblasts.

ER stress, caused by disturbances in normal ER functions [40], is closely related to osteogenesis [23, 24] and has been reported by our group as a vital player in the incidence of OPLL [21]. Studies show that NF-*κ*B can be activated by ER stress under different conditions [41–43]. Herein, we found that the induction of ER stress with EPA elevated USP9X levels, NF-*κ*B nuclei translocation, and ALP activity, which was abolished by PDTC, an NF-*κ*B inhibitor. Reverse results were obtained when ER stress was inhibited by 4-PBA and could abolish the effects of EPA. Thus, we proved that ER stress activates NF-*κ*B and increases USP9X and Cx43 expression, which leads to the promotion of ossification.

To further confirm the results from in vitro study, clinical specimens from OPLL patients were used to examine the expression levels of USP9X, Cx43, and nuclei p65. A significant increase of USP9X, Cx43, and nuclei p65 in OPLL specimens was observed compared with non-OPLL samples. USP9X was positively correlated with CX43 and nuclei p65 in OPLL samples. The results further confirmed in vitro data and provided insight into the role of USP9X, Cx43, and p65 in developing OPLL. Some limitations exist in the present study. Previous studies have suggested that Cx43 can be modified with K63- or K48-linked polyubiquitin chains [44]. Cx43 is also degraded via the endolysosomal and autophagosomal pathways [45]. Which types of ubiquitin chains are linked to Cx43, and whether Cx43 is degraded via endolysosomal and autophagosomal degradation pathways in ligament fibroblast cells remains to be explored in the future. Although our study showed the importance of ER stress-NF*κ*B-USP9X-Cx43 axis in the development of OPLL, further studies are needed to understand USP9X, Cx43-binding proteins, and other factors that might be involved in or contribute to OPLL progression and the development of novel therapeutic approaches for OPLL.

## 5. Conclusion

**Figure 7:**
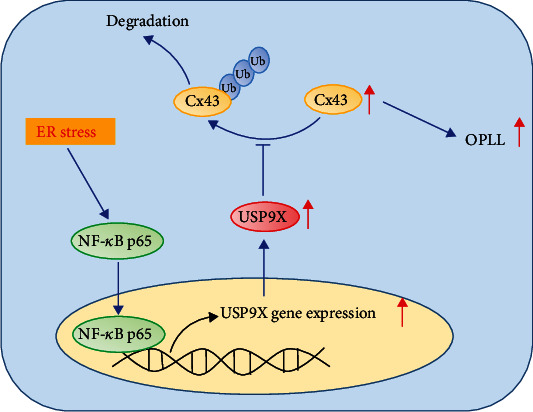
The molecular mechanism by which USP9X-Cx43 regulates the development of OPLL. After being activated by ER stress, NF*κ*B p65 translocated to nuclei and bonded to the promoter region of USP9X to upregulate its transcription. Enhanced expression of USP9X deubiquitinated Cx43 to protect it from degradation and contributed to the development of OPLL.

Taken together, we showed that USP9X, Cx43, and nuclei p65 expression was elevated in OPLL. An enhanced ER stress response activated NF-*κ*B to upregulate USP9X expression. USP9X, working as a deubiquitinase, interacted with Cx43 and stabilized it, leading to upregulation of Cx43, which contributes to the development of OPLL (Figure 7).

## Data Availability

The data of this study are available from the corresponding author for reasonable requests.

## Supplementary Materials

Supplementary Materials Table S1: antibody list. Table S2: primer sequences for real-time PCR. Table S3: target sequences of human USP9X shRNAs. Table S4: identified Cx43-binding proteins by LC-MS. Figure S1 Manipulation of USP9X protein expression in primary ligament fibroblasts. Western blots showed that USP9X was successfully knocked down or over expressed in primary ligament fibroblasts by lentivirus transfection.
