# Supplementary material for: The Effect of the NFκB-USP9X-Cx43 Axis on the Dynamic Balance of Bone Formation/Degradation during Ossification of the Posterior Longitudinal Ligament of the Cervical Spine
Source: Oxid Med Cell Longev. 2022 Mar 29;2022:1604932. doi: 10.1155/2022/1604932 (PMC8983240; doi:10.1155/2022/1604932)
Supplement: Supplementary Materials — Table S1: antibody list. Table S2: primer sequences for real-time PCR. Table S3: target sequences of human USP9X shRNAs. Table S4: identified Cx43-binding proteins by LC-MS. Figure S1 Manipulation of USP9X protein expression in primary ligament fibroblasts. Western blots showed that USP9X was successfully knocked down or over expressed in primary ligament fibroblasts by lentivirus transfection. [file 1604932.f1.docx]

**Table S1.** Antibody list.

| **Primary antibody** | **Company** | **Catalog No.** |
| --- | --- | --- |
| USP9X | Abcam | Ab19879 |
| Flag | Cell Signaling Technology | #14793 |
| Cx43 | Abcam | Ab235583 |
| NF-κB p65 | Cell Signaling Technology | #8242 |
| H3 | Cell Signaling Technology | #4499 |
| Ubiquitin | Abcam | Ab7780 |
| GAPDH | Cell Signaling Technology | #5174 |

**Table S2.** Primer sequences for real-time PCR.

| **Gene** | **Forward primer** | **Reverse primer** |
| --- | --- | --- |
| Cx43 | 5' TTCAAGCCTACTCAACTGC 3' | 5' CTCTTCCTTTCGCATCAC 3' |
| USP9X | 5' CTGCCAGTGATTGGTATG 3' | 5' GAAGGTATTCGGAGAAGC 3' |
| GAPDH | 5' GGATTGTCTGGCAGTAGCC 3' | 5'ATTGTGAAAGGCAGGGAG 3' |

**Table S3.** Target sequences of human USP9X shRNAs.

| **shRNA** | **Target sequence** |
| --- | --- |
| shUXP9X-1 | 5’ GCTTGATCCTTCTCTGTTA 3’ |
| shUXP9X-2 | 5’CCAAGTTACCCATGATCAA3’ |
| shUXP9X-3 | 5’ GCTAGTATTTAGCCCAAAT 3’ |

**Table S4.** Identified Cx43 binding proteins by LC-MS.

| **Accession** | **Description** | **Score** | **Coverage** | **# Proteins** | **# Unique Peptides** | **# Peptides** | **# PSMs** | **# AAs** | **MW [kDa]** | **calc. pI** |
| --- | --- | --- | --- | --- | --- | --- | --- | --- | --- | --- |
| P17302 | GJAL,Gap Junction 43 KDa Heart Protein=Homo sapiens GN=CX43 PE=2 SV=2 - [CX43_HUMAN] | 17458.46 | 47.98 | 2 | 62 | 62 | 1069 | 892 | 98.7 | 8.07 |
| P21333 | Filamin-A OS=Homo sapiens GN=FLNA PE=1 SV=4 - [FLNA_HUMAN] | 5303.60 | 29.22 | 5 | 17 | 18 | 18 | 2647 | 280.6 | 6.06 |
| P12814 | Alpha-actinin-1 OS=Homo sapiens GN=ACTN1 PE=1 SV=2 - [ACTN1_HUMAN] | 4958.99 | 15.47 | 3 | 6 | 11 | 11 | 892 | 103.0 | 5.41 |
| P46821 | Microtubule-associated protein 1B OS=Homo sapiens GN=MAP1B PE=1 SV=2 - [MAP1B_HUMAN] | 4849.45 | 35.92 | 4 | 11 | 11 | 11 | 2468 | 270.5 | 4.81 |
| O75688 | Protein phosphatase 1B OS=Homo sapiens GN=PPM1B PE=1 SV=1 - [PPM1B_HUMAN] | 4677.78 | 40.08 | 2 | 19 | 24 | 189 | 479 | 52.6 | 5.05 |
| Q86WT6 | RING-type E3 ubiquitin transferase TRIM69 OS=Homo sapiens GN=TRIM69 PE=1 SV=1 - [RNF36_HUMAN] | 4524.48 | 68.97 | 3 | 11 | 17 | 145 | 487 | 57.4 | 6.04 |
| P21980 | Protein-glutamine gamma-glutamyltransferase 2 OS=Homo sapiens GN=TGM2 PE=1 SV=2 - [TGM2_HUMAN] | 4515.28 | 20.23 | 2 | 11 | 11 | 12 | 687 | 77.3 | 5.22 |
| P36578 | 60S ribosomal protein L4 OS=Homo sapiens GN=RPL4 PE=1 SV=5 - [RL4_HUMAN] | 4438.48 | 26.93 | 3 | 11 | 11 | 16 | 427 | 47.7 | 11.06 |
| P13533 | Myosin-6 OS=Homo sapiens GN=MYH6 PE=1 SV=5 - [MYH6_HUMAN] | 4251.14 | 40.02 | 5 | 29 | 88 | 207 | 1939 | 223.6 | 5.73 |
| P12883 | Myosin-7 OS=Homo sapiens GN=MYH7 PE=1 SV=5 - [MYH7_HUMAN] | 3823.09 | 30.90 | 5 | 7 | 67 | 159 | 1935 | 223.0 | 5.80 |
| P16403 | Histone H1.2 OS=Homo sapiens GN=HIST1H1C PE=1 SV=2 - [H12_HUMAN] | 3720.96 | 51.64 | 4 | 5 | 16 | 26 | 213 | 21.4 | 10.93 |
| P62736 | Actin, aortic smooth muscle OS=Homo sapiens GN=ACTA2 PE=1 SV=1 - [ACTA_HUMAN] | 3438.38 | 54.91 | 5 | 8 | 21 | 145 | 377 | 42.0 | 5.39 |
| Q9BQA1 | Methylosome protein 50 OS=Homo sapiens GN=WDR77 PE=1 SV=1 - [MEP50_HUMAN] | 3406.31 | 20.76 | 2 | 8 | 8 | 32 | 342 | 36.7 | 5.17 |
| P11142 | Heat shock cognate 71 kDa protein OS=Homo sapiens GN=HSPA8 PE=1 SV=1 - [HSP7C_HUMAN] | 2818.60 | 39.78 | 2 | 25 | 30 | 139 | 646 | 70.9 | 5.52 |
| Q93008 | Probable ubiquitin carboxyl-terminal hydrolase FAF-X OS=Homo sapiens GN=USP9X PE=1 SV=3 - [USP9X_HUMAN] | 2208.15 | 55.14 | 2 | 24 | 16 | 185 | 2570 | 292.1 | 5.80 |
| Q8NES8 | Beta-defensin 124 OS=Homo sapiens GN=DEFB124 PE=2 SV=2 - [DB124_HUMAN] | 2042.61 | 29.58 | 2 | 7 | 2 | 45 | 71 | 8.1 | 8.02 |
| O75385 | ATG1, Serine/threonine-protein kinase ULK1 OS=Homo sapiens GN=ULK1 PE=1 SV=5 - [ULK1_HUMAN] | 1835.17 | 32.80 | 12 | 3 | 23 | 137 | 564 | 60.0 | 8.00 |
| Q9BYW3 | Beta-defensin 126 OS=Homo sapiens GN=DEFB126 PE=2 SV=2 - [DB126_HUMAN] | 1594.97 | 23.42 | 2 | 3 | 3 | 37 | 111 | 12.2 | 9.31 |
| O14744 | Protein arginine N-methyltransferase 5 OS=Homo sapiens GN=PRMT5 PE=1 SV=4 - [ANM5_HUMAN] | 1401.32 | 29.67 | 2 | 26 | 26 | 124 | 637 | 72.6 | 6.29 |
| P35813 | Protein phosphatase 1A OS=Homo sapiens GN=PPM1A PE=1 SV=1 - [PPM1A_HUMAN] | 1392.55 | 14.66 | 1 | 2 | 6 | 49 | 382 | 42.4 | 5.36 |
| P02647 | Apolipoprotein A-I OS=Homo sapiens GN=APOA1 PE=1 SV=1 - [APOA1_HUMAN] | 1253.74 | 76.40 | 1 | 34 | 34 | 75 | 267 | 30.8 | 5.76 |
| Q8WTQ1 | Beta-defensin 104 OS=Homo sapiens GN=DEFB104A PE=2 SV=2 - [D104A_HUMAN] | 1249.18 | 36.11 | 2 | 4 | 2 | 24 | 72 | 8.5 | 9.19 |
| P04114 | Apolipoprotein B-100 OS=Homo sapiens GN=APOB PE=1 SV=2 - [APOB_HUMAN] | 1225.70 | 14.51 | 1 | 62 | 62 | 72 | 4563 | 515.3 | 7.05 |
| P01024 | Complement C3 OS=Homo sapiens GN=C3 PE=1 SV=2 - [CO3_HUMAN] | 1178.14 | 31.81 | 2 | 52 | 52 | 79 | 1663 | 187.0 | 6.40 |
| Q9NXV2 | BTB/POZ domain-containing protein KCTD5 OS=Homo sapiens GN=KCTD5 PE=1 SV=1 - [KCTD5_HUMAN] | 1114.11 | 38.46 | 1 | 10 | 11 | 41 | 234 | 26.1 | 6.24 |
| Q8N687 | Beta-defensin 125 OS=Homo sapiens GN=DEFB125 PE=2 SV=2 - [DB125_HUMAN] | 956.97 | 10.26 | 1 | 2 | 2 | 43 | 156 | 17.5 | 5.49 |
| P52732 | Kinesin-like protein KIF11 OS=Homo sapiens GN=KIF11 PE=1 SV=2 - [KIF11_HUMAN] | 815.13 | 28.50 | 1 | 34 | 34 | 61 | 1056 | 119.1 | 5.64 |
| O15355 | Protein phosphatase 1G OS=Homo sapiens GN=PPM1G PE=1 SV=1 - [PPM1G_HUMAN] | 762.13 | 40.11 | 1 | 19 | 19 | 43 | 546 | 59.2 | 4.36 |
| Q15208 | Serine/threonine-protein kinase 38 OS=Homo sapiens GN=STK38 PE=1 SV=1 - [STK38_HUMAN] | 749.91 | 32.69 | 1 | 18 | 22 | 63 | 465 | 54.2 | 7.15 |
| P38646 | Stress-70 protein, mitochondrial OS=Homo sapiens GN=HSPA9 PE=1 SV=2 - [GRP75_HUMAN] | 697.73 | 34.90 | 1 | 21 | 21 | 37 | 679 | 73.6 | 6.16 |
| P08107 | Heat shock 70 kDa protein 1A/1B OS=Homo sapiens GN=HSPA1A PE=1 SV=5 - [HSP71_HUMAN] | 693.91 | 15.29 | 2 | 6 | 10 | 36 | 641 | 70.0 | 5.66 |
| P09211 | Glutathione S-transferase P OS=Homo sapiens GN=GSTP1 PE=1 SV=2 - [GSTP1_HUMAN] | 635.78 | 58.10 | 1 | 11 | 11 | 36 | 210 | 23.3 | 5.64 |
| P23588 | Eukaryotic translation initiation factor 4B OS=Homo sapiens GN=EIF4B PE=1 SV=2 - [IF4B_HUMAN] | 625.69 | 33.22 | 1 | 19 | 19 | 56 | 611 | 69.1 | 5.73 |
| P78347 | General transcription factor II-I OS=Homo sapiens GN=GTF2I PE=1 SV=2 - [GTF2I_HUMAN] | 619.54 | 26.75 | 3 | 28 | 28 | 48 | 998 | 112.3 | 6.39 |
| P23396 | 40S ribosomal protein S3 OS=Homo sapiens GN=RPS3 PE=1 SV=2 - [RS3_HUMAN] | 608.38 | 54.73 | 1 | 17 | 17 | 47 | 243 | 26.7 | 9.66 |
| Q14681 | BTB/POZ domain-containing protein KCTD2 OS=Homo sapiens GN=KCTD2 PE=1 SV=3 - [KCTD2_HUMAN] | 599.94 | 22.43 | 1 | 3 | 4 | 28 | 263 | 28.5 | 5.29 |
| Q93009 | Ubiquitin carboxyl-terminal hydrolase 7 OS=Homo sapiens GN=USP7 PE=1 SV=2 - [UBP7_HUMAN] | 583.61 | 42.16 | 1 | 15 | 15 | 144 | 1102 | 128.2 | 5.55 |
| P02545 | Prelamin-A/C OS=Homo sapiens GN=LMNA PE=1 SV=1 - [LMNA_HUMAN] | 580.41 | 23.80 | 1 | 15 | 15 | 27 | 664 | 74.1 | 7.02 |
| P02671 | Fibrinogen alpha chain OS=Homo sapiens GN=FGA PE=1 SV=2 - [FIBA_HUMAN] | 573.70 | 28.29 | 1 | 24 | 24 | 51 | 866 | 94.9 | 6.01 |
| P25705 | ATP synthase subunit alpha, mitochondrial OS=Homo sapiens GN=ATP5A1 PE=1 SV=1 - [ATPA_HUMAN] | 545.01 | 34.90 | 1 | 21 | 21 | 32 | 553 | 59.7 | 9.13 |
| P78371 | T-complex protein 1 subunit beta OS=Homo sapiens GN=CCT2 PE=1 SV=4 - [TCPB_HUMAN] | 508.20 | 26.92 | 1 | 15 | 15 | 28 | 535 | 57.5 | 6.46 |
| P06576 | ATP synthase subunit beta, mitochondrial OS=Homo sapiens GN=ATP5B PE=1 SV=3 - [ATPB_HUMAN] | 496.99 | 38.19 | 1 | 15 | 15 | 31 | 529 | 56.5 | 5.40 |
| P68104 | Elongation factor 1-alpha 1 OS=Homo sapiens GN=EEF1A1 PE=1 SV=1 - [EF1A1_HUMAN] | 491.93 | 18.61 | 3 | 11 | 11 | 39 | 462 | 50.1 | 9.01 |
| P01023 | Alpha-2-macroglobulin OS=Homo sapiens GN=A2M PE=1 SV=3 - [A2MG_HUMAN] | 488.72 | 18.52 | 2 | 24 | 24 | 35 | 1474 | 163.2 | 6.46 |
| Q7KZF4 | Staphylococcal nuclease domain-containing protein 1 OS=Homo sapiens GN=SND1 PE=1 SV=1 - [SND1_HUMAN] | 454.62 | 16.81 | 1 | 5 | 5 | 6 | 910 | 101.9 | 7.17 |
| Q14315 | Filamin-C OS=Homo sapiens GN=FLNC PE=1 SV=3 - [FLNC_HUMAN] | 428.06 | 12.53 | 1 | 4 | 5 | 5 | 2725 | 290.8 | 5.97 |
| P49588 | Alanine--tRNA ligase, cytoplasmic OS=Homo sapiens GN=AARS PE=1 SV=2 - [SYAC_HUMAN] | 383.85 | 22.58 | 1 | 2 | 2 | 2 | 968 | 106.7 | 5.53 |
| P09874 | Poly [ADP-ribose] polymerase 1 OS=Homo sapiens GN=PARP1 PE=1 SV=4 - [PARP1_HUMAN] | 352.62 | 32.76 | 1 | 2 | 2 | 2 | 1014 | 113.0 | 8.88 |
| Q13263 | Transcription intermediary factor 1-beta OS=Homo sapiens GN=TRIM28 PE=1 SV=5 - [TIF1B_HUMAN] | 349.71 | 12.16 | 1 | 2 | 2 | 2 | 835 | 88.5 | 5.77 |
| O75746 | Calcium-binding mitochondrial carrier protein Aralar1 OS=Homo sapiens GN=SLC25A12 PE=1 SV=2 - [CMC1_HUMAN] | 336.31 | 24.13 | 1 | 2 | 3 | 3 | 678 | 74.7 | 8.38 |
| P35542 | Serum amyloid A-4 protein OS=Homo sapiens GN=SAA4 PE=1 SV=2 - [SAA4_HUMAN] | 326.10 | 16.15 | 1 | 2 | 2 | 3 | 130 | 14.7 | 9.07 |
| O95400 | CD2 antigen cytoplasmic tail-binding protein 2 OS=Homo sapiens GN=CD2BP2 PE=1 SV=1 - [CD2B2_HUMAN] | 321.06 | 17.04 | 1 | 2 | 2 | 2 | 341 | 37.6 | 4.61 |
| Q3V6T2 | Girdin OS=Homo sapiens GN=CCDC88A PE=1 SV=2 - [GRDN_HUMAN] | 315.86 | 21.02 | 1 | 2 | 2 | 2 | 1871 | 215.9 | 6.21 |

**
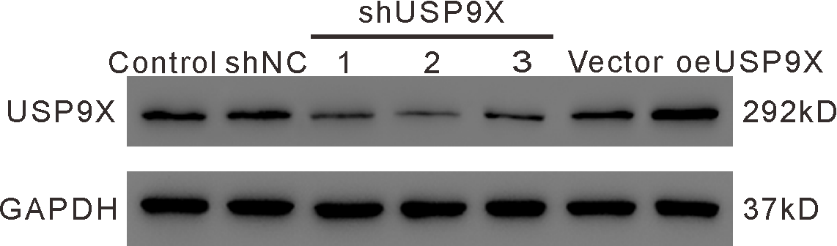
**

**Figure S1.** Manipulation of USP9X protein expression in primary ligament fibroblasts. Western blots showed that USP9X was successfully knocked down or over expressed in primary ligament fibroblasts by lentivirus transfection.
